# Does endodontic treatment modify serum inflammatory markers of cardiovascular risk in individuals with asymptomatic apical periodontitis? a systematic review and meta-analysis

**DOI:** 10.1007/s00784-026-06857-0

**Published:** 2026-05-02

**Authors:** Randerson Silva Araújo, Erika Bárbara Abreu Fonseca Thomaz, Elma Izze da Silva Magalhães, Juliana Balbinot Hilgert, Soraia de Fátima Carvalho Souza

**Affiliations:** 1 Postgraduate Program in Dentistry, Federal University of Maranhão (UFMA), São Luís, Maranhão, Brazil; 2 Department of Public Health, Federal University of Maranhão (UFMA), São Luís, Maranhão, Brazil; 3 Department of Public Health, Federal University of Santa Maria (UFSM), Santa Maria, Rio Grande do Sul Brazil; 4 Department of Preventive and Social Dentistry, Graduate Program in Dentistry, Federal University of Rio Grande do Sul (UFRGS), Porto Alegre, Rio Grande do Sul Brazil

**Keywords:** Apical Periodontitis, Inflammation, Inflammatory Mediators, Heart Disease Risk Factors, Root Canal Therapy

## Abstract

**Objective:**

Plasma inflammatory biomarkers linked to cardiovascular risk have been associated with asymptomatic apical periodontitis. However, it remains unclear whether endodontic treatment can reverse these alterations. This review evaluated the effect of endodontic treatment on inflammatory markers in individuals with asymptomatic apical periodontitis.

**Materials and methods:**

A comprehensive search was conducted in PubMed/Medline, Embase, Web of Science, Scopus, VHL, gray literature, and reference lists between October and November 2022, with an update in September 2025. Risk of bias was assessed using the Newcastle–Ottawa Scale, and the certainty of evidence using the GRADE approach. Random-effects meta-analysis estimated pooled mean differences (MD) and 95% confidence intervals (95%CI) for serum inflammatory markers concentrations between treated individuals and controls (α = 5%).

**Results:**

The search identified 6,295 records; sixteen studies were assessed and eight included in the quantitative synthesis. All studies showed moderate risk of bias, and evidence certainty was very low. Meta-analysis suggested possible reductions in C-reactive protein (CRP) [MD = 0.76 (95% CI: − 0.15, 1.67)] , interleukin-6 (IL-6) [MD = 0.81 (95% CI:–0.27, 1.90)], and tumor necrosis factor-alpha (TNF-α) [MD = 1.04 (95% CI:–0.38, 2.46)] after endodontic treatment, with levels similar to control groups.

**Conclusion:**

Evidence, although limited, suggests endodontic treatment may lower serum CRP, IL-6, and TNF-α levels in asymptomatic apical periodontitis patients.

**Clinical relevance:**

Endodontic treatment of asymptomatic apical periodontitis may help reduce systemic inflammatory biomarkers associated with cardiovascular risk, reinforcing its potential role beyond local infection control.

**Supplementary Information:**

The online version contains supplementary material available at 10.1007/s00784-026-06857-0.

## Introduction

Cardiovascular diseases are the leading cause of mortality worldwide, accounting for 32% of all annual deaths [1]. The development is strongly associated with behavioral risk factors such as smoking, excessive alcohol intake, unhealthy diets, and physical inactivity, which trigger metabolic changes including hypertension, dyslipidemia, overweight, and obesity [2]. A chronic low-grade systemic inflammatory state, characterized by increased serum inflammatory markers, also contributes significantly to the pathogenesis of cardiovascular diseases, especially atherosclerosis—recognized as a major predictor of cardiovascular risk [3].

In this context, asymptomatic apical periodontitis, a chronic and highly prevalent inflammatory condition affecting the periapical tissues [4], has been associated with elevated systemic levels of pro-inflammatory cytokines, including interleukin-6 (IL-6), interleukin-1β (IL-1β), interleukin-18 (IL-18), tumor necrosis factor-alpha (TNF-α), and C-reactive protein (CRP) [5, 6]. The increase in these inflammatory mediators may contribute to the maintenance of a chronic low-grade systemic inflammatory state, which represents one of the proposed mechanisms linking endodontic diseases to alterations associated with increased cardiovascular risk [7, 8]. More broadly, the biological plausibility of this association has been explained through three main pathways [9]: (a) the occurrence of transient bacteremia originating from the infected root canal system [10, 11]; (b) the vascular dissemination of toxins and bacterial byproducts derived from endodontic microorganisms [12]; and (c) the systemic low-grade inflammatory response associated with oxidative stress resulting from the persistence of chronic apical periodontitis [7, 8, 13].

Chronic periapical inflammation is mediated by a complex innate and adaptive immune response involving both pro- and anti-inflammatory activities [14–16]. Immune system cells such as macrophages, lymphocytes, and leukocytes are recruited to the periapical region, promoting the release of inflammatory mediators including chemokines and cytokines. The result is progressive periapical tissue damage, such as apical bone resorption [17, 18], regulated by pro-inflammatory cytokines—IL-1β, IL-6, interferon-gamma (IFN-γ), and TNF-α—and by anti-inflammatory cytokines such as transforming growth factor-beta (TGF-β) and interleukin-4 (IL-4) [19].

Prior to 2019, systematic reviews and meta-analyses found no significant reduction in serum CRP following endodontic treatment for individuals with asymptomatic apical periodontitis. Although several inflammatory markers had been investigated, only CRP presented sufficient data for effect estimation, and the high methodological heterogeneity among studies made it impossible to confirm the efficacy of endodontic treatment in modulating this inflammatory biomarker [7, 8]. However, a more recent meta-analysis reported divergent and more promising results: after six months of follow-up, a significant decrease in serum CRP levels was observed in individuals with asymptomatic apical periodontitis who underwent endodontic treatment, suggesting possible systemic anti-inflammatory benefits [20].

Given the inconclusive findings, the impact of endodontic treatment on the modulation of serum levels of inflammatory mediators associated with cardiovascular risk remains uncertain. Therefore, the aim of this study was to critically evaluate the effect of endodontic treatment on serum levels of inflammatory markers among healthy individuals with asymptomatic apical periodontitis.

## Methods

This systematic review and meta-analysis was conducted in accordance with the recommendations of the *Preferred Reporting Items for Systematic Reviews and Meta-Analyses* (PRISMA, 2020) [21] and registered in the *International Prospective Register of Systematic Reviews* (PROSPERO; CRD42023391374), available at (https://www.crd.york.ac.uk/Prospero/*).*

### Eligibility Criteria

#### Inclusion Criteria

Observational (cohort and case-control) and experimental studies (randomized and non-randomized clinical trials, including before-and-after studies) that evaluated the effect of endodontic treatment in participants with asymptomatic apical periodontitis were included, without restrictions on age, ethnicity, sex, or socioeconomic status. These study designs were selected because they included comparison group and/or longitudinal design, allowing appropriate analysis of the effect of the intervention/exposure on the outcomes.

#### Exclusion Criteria

Cross-sectional studies, case reports, expert opinions, technical reports, letters to the editor, and all types of reviews were excluded. Studies that did not include serum measurements of inflammatory markers before and after endodontic intervention, or those including individuals with acute endodontic conditions (e.g., acute dentoalveolar abscess), comorbidities, or exposure to uncontrolled behavioral risk factors were also excluded.

### Search Strategy and Databases

The systematic search was conducted independently and blindly by two reviewers (RSA and EISM), based on the PICO strategy: population [(P) – individuals with apical periodontitis], intervention [(I) – endodontic treatment], comparison [(C) – post-treatment values and external control of healthy individuals], and outcome [(O) – inflammatory markers of cardiovascular risk, cytokines, and biomarkers]. *MeSH* (Medical Subject Headings), *DeCS* (Health Sciences Descriptors), and equivalent *Emtree* terms were combined using Boolean operators (OR and AND) to ensure search comprehensiveness. Detailed search strategies for each database are provided in Supplementary Tables S1–S6.

The search was initially conducted between October and November 2022 and updated in September 2025, with no restrictions on language or publication date. Databases searched included: PubMed/MEDLINE, Embase, Web of Science, Scopus, and VHL (MEDLINE, IBECS, BBO, LILACS). Grey literature databases included the CAPES Theses and Dissertations Catalog, the Brazilian Digital Library of Theses and Dissertations, ProQuest, and Google Scholar. Reference lists of relevant articles were also reviewed.

### Study Selection

Search results were imported into *EndNote Web* (Clarivate Analytics, USA) for duplicate removal. Subsequently, study titles and information were organized in a *Microsoft Excel®* 2022 (Microsoft Corporation, Washington, USA) spreadsheet and rechecked for duplicates. Titles and abstracts were screened independently by two reviewers (RSA and EISM) according to the eligibility criteria. Discrepancies were resolved by a third reviewer (SFCS). Only eligible studies were read in full text.

### Data Extraction

Data extraction was performed independently by two reviewers (RSA and EISM) under senior supervision (SFCS and EBAFT). Data were extracted and tabulated using a standardized data collection form developed by consensus. Extracted data included: authors, year of publication, country, study design, population, sample size, age range, type of endodontic treatment, inflammatory marker evaluated, means and standard deviations of inflammatory markers before and after endodontic treatment, 95% confidence intervals (95% CI), follow-up duration, confounders, presence and type of control group, means and standard deviations of inflammatory markers in control groups, main findings, and reported limitations. Study authors were contacted for missing or unclear information.

### Risk of Bias and Quality of Evidence Assessment

Risk of bias was assessed using the *Newcastle–Ottawa Scale* (NOS) for cohort studies adapted for intervention studies, independently by two examiners (RSA and EISM) [22, 23]. Studies scoring 7–9 stars were classified as low risk of bias, 4–6 stars moderate risk, and 0–3 stars high risk. Discrepancies were resolved by consensus. The quality of the evidence was evaluated using the *Grading of Recommendations Assessment*,* Development and Evaluation* (GRADE) system.

### Statistical Analysis

Meta-analyses estimated pooled mean differences (MD) and 95% confidence intervals (95% CI) of inflammatory marker concentrations between groups undergoing endodontic treatment and controls (self-controlled or external). Negative MD values or 95% CI including zero indicated reductions in inflammatory markers after endodontic treatment to levels similar to those of healthy controls without apical periodontitis.

Heterogeneity was assessed using the Cochran’s Q-test and I² statistic. Due to the variability across study designs, populations, and interventions, a random-effects model with the Mantel–Haenszel estimation method was applied [24]. Subgroup analyses compared studies with self-control groups and those with external controls (Supplementary Fig. 1–3).

Because fewer than 10 studies were included, publication bias assessment using Egger’s test or funnel plot was not perfomed. All statistical analyses were performed using *Stata* version 14.0 (StataCorp LP, College Station, TX, USA), adopting a 5% alpha level.

## Results

### Search Results

**Fig. 1:**
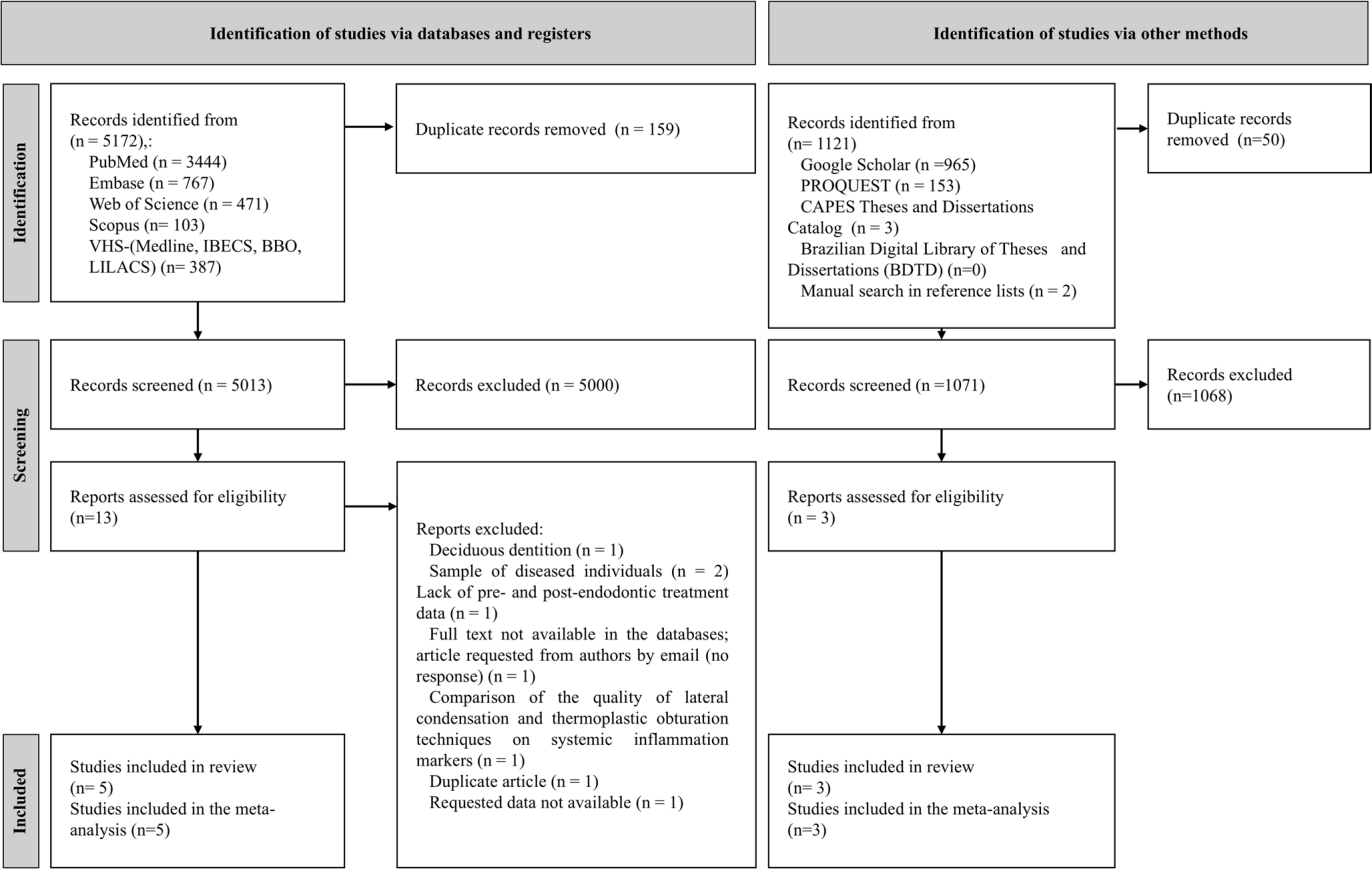
PRISMA Flow Diagram. Process of identification and selection of studies included in the systematic review and meta-analysis

A total of 6,295 publications were identified across all databases searched. After duplicate removal and screening of titles and abstracts, 16 publications were selected for full-text reading, two of which were identified through manual reference screening [25, 26]. At this stage, eight publications were excluded, reasons for exclusion are detailed in Fig. 1. Eight studies were included in the qualitative and quantitative synthesis.

### Study Characteristics

Table 1 summarizes the main methodological characteristics of the included studies. Four studies presented a hybrid design— “before-and-after” studies with a cross-sectional control group of healthy individuals; two were “before-and-after” studies without an internal control group; and two were preliminary intervention studies also without internal controls. Studies were conducted in Brazil (*n* = 1), Chile (*n* = 1), the United States (*n* = 1), Italy (*n* = 1), India (*n* = 3), and the United Kingdom (*n* = 1). Sample sizes ranged from 11 to 115 participants, aged 18 to 75 years. Twenty-three inflammatory markers were evaluated, with C-reactive protein (CRP) (*n* = 7), IL-1 (*n* = 2), IL-6 (*n* = 2), TNF-α (*n* = 2), intercellular adhesion molecule-1 (ICAM-1) (*n* = 2), vascular cell adhesion molecule-1 (VCAM-1) (*n* = 2), and complement component 3 (C3) (*n* = 2), being the most frequently assessed.

**Table 1:** Qualitative summary of the studies included in the systematic review

Author/Year	Country	Study Design	Sample Size	Age Range	Type of Intervention	Control Group	Cardiovascular Risk Biomarkers	Follow-up	Main Findings	Limitations
Marton & Kiss, 1992 [25]	United States of America	Before-and-after clinical study	*n* = 36	Mean age of men = 28.1 years Mean age of women = 25.2 years	Conventional endodontic treatment with surgical complement	No internal control group	AAT AMG C3 CRP HPT IgA IgG IgM	3 months	Serum concentrations of the evaluated markers decreased after 3 months of endodontic treatment with surgical complement, particularly CRP.	Small sample size and absence of a control group.
Brosco, 2009 [31]	Brazil	Before-and-after clinical study	*n* = 26	23–40 years	Conventional endodontic treatment(*n* = 13)	Internal control group of healthy patients (*n* = 13)	CRP	30 days	No statistically significant differences were observed in CRP values before and after endodontic treatment.	Small sample size
Bergandi et al., 2019 [32]	Italy	Before-and-after clinical study	*n* = 43	< 35 years	Conventional endodontic treatment (*n* = 23)	Internal control group of healthy patients (*n* = 20)	TNF-α IL-6 IL-1 sCD14 Endothelin-1 ICAM-1 VCAM-1 E-selectin	12 months	After treatment, serum concentrations of IL-1, sCD14, ET-1, ICAM-1, and E-selectin significantly decreased, becoming comparable to those of control individuals.	Small sample size
Bains et al., 2020 [26]	India	Before-and-after clinical study	*n* = 11	Mean age = 21.1 years	Conventional endodontic treatment	No internal control group	CRP	1 month	Serum CRP concentrations significantly decreased one month after endodontic intervention.	Small sample size and absence of a control group.
Poornima et al., 2021 [6]	India	Preliminary prospective longitudinal interventional study	*n* = 15	20–40 years	Conventional endodontic treatment	No internal control group	CRP	6 months	After 6 months of follow-up, there was a significant reduction in mean hs-CRP levels.	Small sample size and absence of a control group.
Bakhsh et al., 2022 [27]	United Kingdom	Cohort study	*n* = 115	19–75 years	Endodontic retreatment(*n* = 35) Periapical surgery (*n* = 30)	Internal control group of healthy patients (*n* = 50)	IL-1β IL-6 IL-8 TNF-α Pentraxin 3 ICAM-1 VCAM-1 CRP FGF-23 MMP-2 MMP-8 MMP-9 C3 ADMA	12 months	After 3 and 6 months, an increase in some inflammatory markers such as CRP and IL-1 was observed; however, after 12 months, these markers decreased to levels similar to those of the controls.	Groups with small sample size.
Kumar et al., 2022 [28]	India	Prospective interventional study	*n* = 50	18–40 years	Conventional endodontic treatment(*n* = 25)	Internal control group of healthy patients (*n* = 25)	CRP	6 months	After 6 months of follow-up, there was a significant reduction in mean hs-CRP levels.	Groups with small sample size.
Garrido et al., 2024 [33]	Chile	Before-and-after clinical study	*n* = 29	16–40 years	Conventional endodontic treatment	No internal control group	CRP TNF-α IL-6 IL-10 IL-1β E- selectin	6 months	In individuals with apical periodontitis and cardiovascular risk (CRP ≥ 1 mg/L), both CRP and its monomeric isoform significantly decreased at 1 and 6 months.	Small sample size and absence of a control group.

*IL-1β* interleukin-1 beta, *IL-6* interleukin-6, *IL-8* interleukin-8, *TNF-α* tumor necrosis factor-alpha, *ICAM-1* intercellular adhesion molecule-1, *VCAM-1* vascular cell adhesion molecule-1, *CRP* C-reactive protein, *FGF-23* fibroblast growth factor-23, *MMP-2* matrix metalloproteinase-2, *MMP-8* matrix metalloproteinase-8, *MMP-9* matrix metalloproteinase-9, *C3* complement component 3, *ADMA* asymmetric dimethylarginine, *sCD14* soluble cluster of differentiation 14, *ET-1* endothelin-1, *AAT* alpha-1 antitrypsin, *AMG* alpha-2 macroglobulin, *HPT* haptoglobin, *IgA* immunoglobulin A, *IgG* immunoglobulin G, *IgM* immunoglobulin

The endodontic treatment protocols varied: six studies used conventional nonsurgical endodontic treatment, one combined nonsurgical treatment with surgical intervention, and one compared nonsurgical endodontic retreatment with apical microsurgery. Follow-up periods ranged from 7 days to 12 months.

### Risk of Bias and Quality of Evidence Assessment

According to the Newcastle–Ottawa Scale (NOS), all studies presented a moderate risk of bias (Table 2). Based on the GRADE system, the quality of evidence for the outcomes included in the meta-analysis was rated as very low, mainly due to the nonrandomized design of the studies, the impossibility of random allocation of participants, and the heterogeneity and imprecision of the results (Table 3).

**Table 2:** Assessment of the methodological quality of the studies according to the NOS criteria*

Author/Year	Selection				Comparability	Outcome		
	1.	2.	3.	4.	1.	1.	2.	3.
Márton & Kiss, 1992 [25]			★		★	★	★	
Brosco, 2009 [31]			★	★	★★	★	★	
Bergandi et al., 2019 [32]			★	★﻿	★	★	★	★
Bains et al., 2020 [26]			★	★	★	★	★	
Poornima et al., 2021 [6]			★	★		★	★	★
Bakhsh et al., 2022 [27]			★	★	★★	★	★	
Kumar et al., 2022 [28]			★	★	★	★	★	★
Garrido et al., 2024 [33]			★	★	★	★	★	

*Adapted for interventional studies. Rating scale: 7 to 9 stars = low risk of bias; 4 to 6 stars = moderate risk of bias; 0 to 3 stars = high risk of bias

Selection: 1. Representativeness of the sample; 2. Selection of the non-exposed cohort; 3. Ascertainment of exposure; 4. Outcome of interest not present at the start of the study. Comparability:1.Comparability of cohorts based on study design or analysis for controlling confounding factors. Outcome: 1. Assessment of outcome; 2. Was follow-up long enough for outcomes to occur? 3. Adequacy of cohort follow-up. A study may receive a maximum of one star (★) for each numbered item in the Selection and Outcome categories. A maximum of two stars (★★) may be awarded for Comparability

**Table 3:** Grading of recommendations assessment, development and evaluation (GRADE)

Certainty assessment							№ of patients		Effect		Certainty	Importance
№ of studies	Study design	Risk of bias	Inconsistency	Indirectness	Imprecision	Other considerations	Endodontic treatment	Healthy controls or post-treatment measures	Relative (95% CI)	Absolute (95% CI)		
7	non-randomised studies	not serious	serious^a^	not serious	serious^b^	dose response gradient	194	188	-	SMD **0.76 SD more** (0.15 fewer to 1.67 more)	⨁◯◯◯ Very low^a, b^	Critical
2	non-randomised studies	not serious	serious^c^	not serious	serious^d^	none	88	60	-	SMD **6.26 SD higher** (4.65 higher to 7.87 higher)	⨁◯◯◯ Very low^c, d^	Critical
2	non-randomised studies	not serious	serious^e^	not serious	serious^f^	none	88	38	-	SMD **0.03 SD higher** (0.15 lower to 0.21 higher)	⨁◯◯◯ Very low^e, f^	Critical
2	non-randomised studies	not serious	serious^g^	not serious	serious^h^	none	88	56	-	SMD **7.26 SD higher** (3.61 higher to 10.91 higher)	⨁◯◯◯ Very low^g, h^	Critical
2	non-randomised studies	not serious	serious^i^	not serious	serious^j^	none	88	38	-	SMD **44102.04 SD lower** (122551.39 lower to 34347.32 higher)	⨁◯◯◯ Very low^i, j^	Critical

*CRP* C-reactive protein, *IL-6* interleukin-6, *VCAM-1* vascular cell adhesion molecule-1, *TNF-α* tumor necrosis factor alpha, *ICAM-1* intercellular adhesion molecule-1. *CI* Confidence interval, *SMD* Standardised mean difference. ^a, c,e, g,i^: I^2^ > 50%, with a significant p-value. ^b, d,f, h,j^: total *n* <400

### Meta-Analysis

Eight studies assessing serum levels of inflammatory markers associated with cardiovascular risk before and after endodontic intervention were included in the meta-analyses. For one study, authors were contacted, and the raw data were made available [27].

Among the studies with control groups (*n* = 4; Table 1), all used a healthy population as external controls. For the four studies without controls, external controls were used based on two approaches: (1) values from the control group of another study included in this review [28]; and (2) reference values from a healthy population for CRP, IL-6, and TNF-α [29], as well as for the cell adhesion molecules ICAM-1 and VCAM-1 [30]. This strategy allowed the inclusion of all studies in quantitative analyses.

**Fig. 2:**
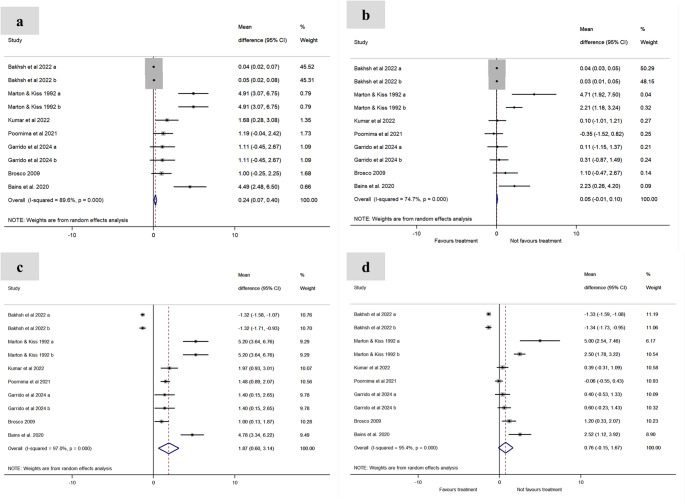
Effect of endodontic treatment on serum levels of CRP (**a–d).** **a**. Baseline with internal control group; **b**. Post-treatment with internal control group; **c**. Baseline with external control group; **d**. Post-treatment with external control group.

**Fig. 3:**
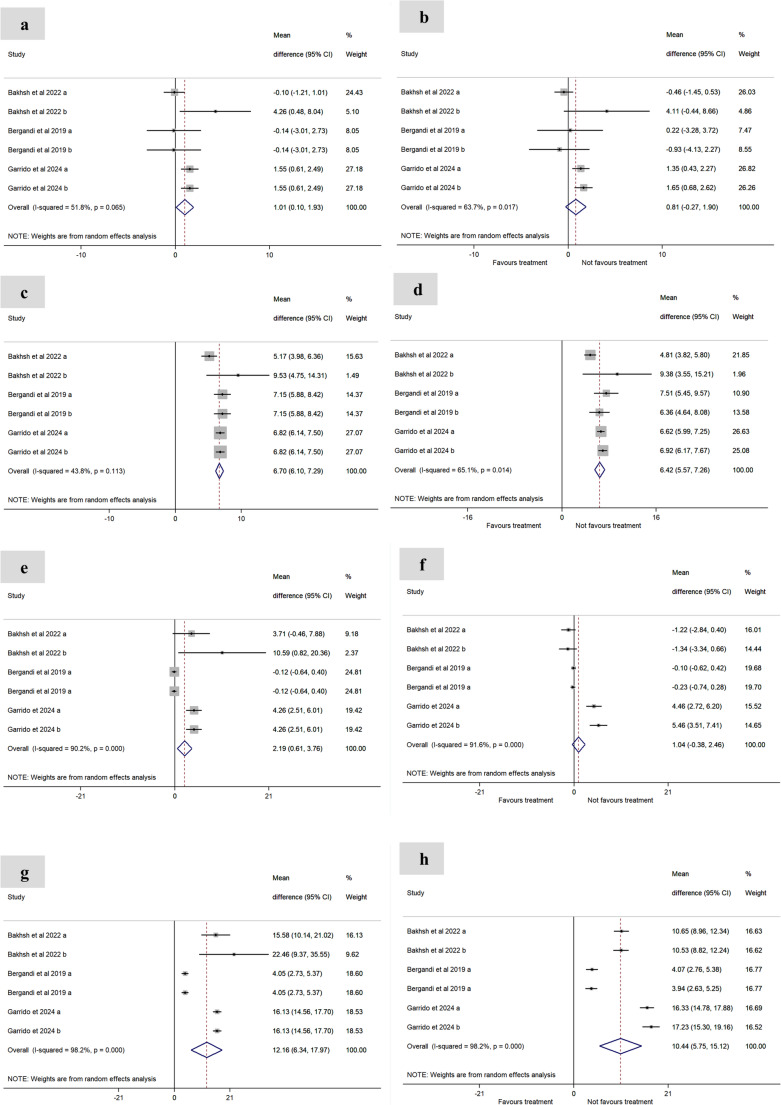
Effect of endodontic treatment on serum levels of IL-6 (**a–d**) and TNF-α (**e–h**).IL-6: **a**. Baseline with internal control group; **b**. Post-treatment with internal control group; **c**. Baseline with external control group; **d**. Post-treatment with external control group. TNF-α: **e**. Baseline with internal control group; **f**. Post-treatment with internal control group; **g**. Baseline with external control group; **h**. Post-treatment with external control group

The pooled mean differences (MD) and their corresponding 95% confidence intervals (95% CI) revealed that endodontic treatment reduced inflammatory markers: CRP [MD = 0.05 (95% CI: − 0.01, 0.10), self-control – Fig. 2b; MD = 0.76 (95% CI: − 0.15, 1.67), external control – Fig. 2 d] . ; IL-6 [MD = 0.81 (95% CI: − 0.27, 1.90), self-control – Fig. 3b]; and TNF-α [MD = 1.04 (95% CI: − 0.38, 2.46), self-control – Fig. 3f], with values approaching control group levels.

**Fig. 4:**
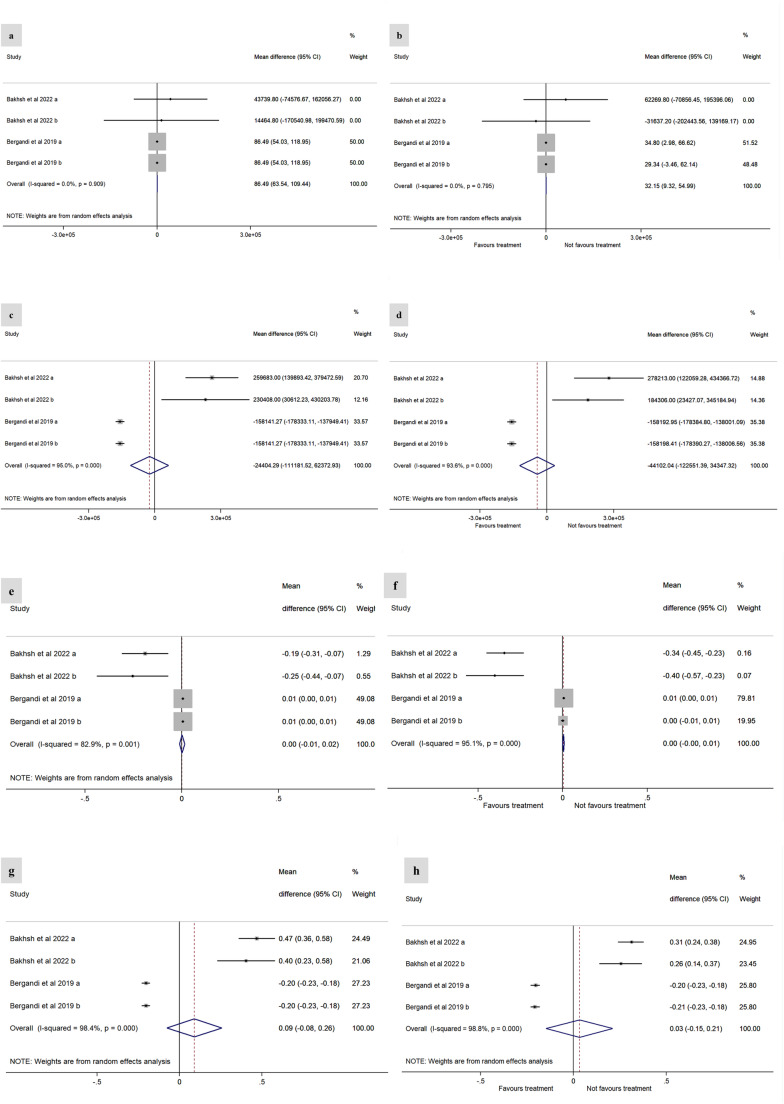
Effect of endodontic treatment on serum levels of ICAM-1 ( **a**– **d**) and VCAM-1 ( **e**– **h**). ICAM-1: **a**. Baseline with internal control group. **b**. Post-endodontic treatment with internal control group. **c**. Baseline with external control group. **d**. Post-endodontic treatment with external control group. VCAM-1: **e**. Baseline with internal control group; **f**. Post-treatment with internal control group; **g**. Baseline with external control group; **h**. Post-treatment with external control group

No differences were observed for ICAM-1 [MD = 32.15 (95% CI: 9.32, 54.99), self-control – Fig. 4b; MD = − 44102.04 (95% CI: − 122551.39, 34347.32), external control – Fig. 4 d] and VCAM-1 [MD= 0.00 (95% CI: - 0.00, 0.01), self-control - Fig. 4f; MD= 0.03 (95% CI: -0.15, 0.21), external control - Fig. 4h] between groups before or after intervention.

In all analyses, I² values were greater than 50% and p-values were below 0.05, indicating the presence of heterogeneity, which persisted even after subgroup analyses (Supplementary Fig. 1–3).

## Discussion

Asymptomatic apical periodontitis has been associated with increased serum levels of inflammatory markers, raising interest in investigating whether endodontic treatment could reverse these elevations. This systematic review and meta-analysis synthesized the available evidence on the effect of endodontic treatment on serum levels of inflammatory markers associated with cardiovascular risk prediction. The analyses suggest a possible reduction in CRP, IL-6, and TNF-α levels, indicating a potential systemic benefit resulting from endodontic treatment.

These findings gain relevance since evidence has increasingly demonstrated the role of chronic low-grade systemic inflammation in the development and progression of cardiovascular conditions. Over the past three decades, several studies have shown that infections of endodontic origin can influence systemic health [34–40], contributing to elevated plasma levels of various inflammatory markers, including IL-1β, IL-6, CRP, TNF-α, and adhesion molecules, among others [5–8].

CRP, secreted by hepatocytes in response to cytokine stimulation such as IL-1, IL-6, and TNF-α, is the main biomarker capable of predicting the risk of developing cardiovascular events, being correlated with endothelial dysfunction and various stages of coronary artery disease [39]. In this review, individuals with asymptomatic apical periodontitis exhibited higher baseline mean serum CRP levels compared with controls, corroborating findings from other studies [26–28, 33], with a tendency toward reduction to similar levels following endodontic treatment. Garrido et al. (2019) also reported a significant association between the presence of at least one periapical lesion and cardiovascular risk in individuals with serum CRP concentrations above 3 mg/L. This risk increased by 3.3-fold in the presence of a second periapical lesion, reinforcing the potential link between oral infections and systemic health [05].

Until 2019, previous meta-analyses [7, 8] did not find significant reductions in blood CRP levels before and after endodontic treatment. However, more recent studies, such as that by Jakovljevic et al. (2024), found that after six months of follow-up, individuals with asymptomatic apical periodontitis who underwent endodontic intervention showed significantly reduced serum CRP levels—a result corroborated by the findings of this study. Although promising, this evidence should be interpreted with caution, as CRP levels can be influenced by multiple factors. Moreover, uninvestigated and poorly understood underlying mechanisms may also contribute to this association. These results support the hypothesis that well-executed endodontic treatment may play a contributory role in reducing systemic inflammation, improving overall health in individuals with asymptomatic apical periodontitis, and potentially influencing cardiovascular risk classification according to the American Heart Association, which defines three categories based on serum levels: low risk (< 1 mg/L), intermediate risk (1–3 mg/L), and high risk (> 3 mg/L) [41].

 The VCAM-1 promotes endothelial dysfunction and leukocyte migration [42, 43]. Elevated VCAM-1 levels are associated with the severity and complications of coronary artery disease [44–46] and can indicate the presence of early chronic inflammatory states, serving as a marker to predict subclinical atherosclerosis and cardiovascular mortality rates [47, 48]. In our study, VCAM-1 levels remained unchanged after endodontic treatment, with no diferences observed between groups at baseline or after the intervention. 

Meta-analysis with self-controlled designs showed statistically significant baseline differences in IL-6 levels between healthy individuals and those with asymptomatic apical periodontitis, with significant reduction following endodontic treatment, corroborating the findings of Georgiou et al. (2019). However, this difference was no longer evident when an external control group was applied. In healthy individuals, IL-6 can be produced by adipocytes and released during muscle contraction [49, 50], which explains its circulating presence even in the absence of inflammation. Furthermore, its levels are modulated by physical activity [51]and increase with age, participating in processes such as tissue regeneration, metabolism, and bone homeostasis [52].

Significant differences in serum TNF-α levels were also observed before and after endodontic treatment. In bone-related diseases, TNF-α induces osteoclast recruitment, promoting excessive bone loss, as seen in rheumatoid arthritis, periodontal disease, and periapical lesions [53]. In periapical interstitial fluid, intense TNF-α activity may contribute to altered plasma levels of this mediator in individuals with asymptomatic apical periodontitis [19]. Following endodontic intervention, these values decreased to levels similar to those of healthy controls. Despite this evidence, the confidence in the results remains limited, as the quality of evidence according to the GRADE system was rated very low. This limitation is mainly due to the high heterogeneity (I² > 50%), differences in sample sizes, study designs, small overall participant numbers, age variations, and differences among endodontic interventions (non-surgical and surgical endodontic treatment, and non-surgical and surgical retreatment).

ICAM-1, induced by inflammatory cytokines in endothelial, epithelial, and immune cells is elevated in chronic obstructive pulmonary disease, asthma, sepsis, atherosclerosis, and cancer, whereas its serum levels in healthy individuals remain low [30]. Although Bergandi et al. (2019) observed higher ICAM-1 levels in patients with apical periodontitis, this meta-analysis did not confirm these findings.

Some limitations of the studies included in this systematic review and meta-analysis should be considered when interpreting the results: (1) small sample sizes and non-representative populations, limiting generalizability; (2) lack of standardization in follow-up duration and age ranges; (3) variability in the inflammatory markers assessed; (4) high heterogeneity among studies, even after subgroup analyses; and (5) few studies to assess publication bias. Included studies had moderate risk of bias according to the Newcastle–Ottawa Scale (NOS) criteria, which exhibit validity comparable to the Cochrane ROBINS-I tool [23].

A major strength is the control of potential confounding bias: the included studies selected samples while minimizing confounders by excluding systemically compromised patients, individuals with chronic oral infections, and recent anti-inflammatory and/or antibiotic use within months prior to blood collection. Additionally, in the meta-analyses, the imputation of healthy external controls reinforced the consistency of the estimates [28]. This was feasible because, in all included studies, individuals in control groups were systemically healthy. Moreover, the robustness was confirmed by meta-analysis considering reference values for healthy individuals without systemic conditions.

These findings highlight the need for further research on the topic. Oral diseases have been recognized as a neglected epidemic [54], and it is estimated that approximately 75% of untreated caries cases occur in middle-income countries [55], contributing to the high prevalence of asymptomatic apical periodontitis—a condition affecting at least one tooth in approximately half of the global population [56].

Given this scenario, understanding the impact of patients’ endodontic health on overall health promotion is crucial. This recognition, especially among individuals at high risk for chronic noncommunicable diseases, emphasizes the need for an integrated approach to healthcare delivery [1, 27].

Future longitudinal studies with larger samples, standardized follow-up periods, and investigations into the molecular mechanisms underlying chronic low-grade systemic inflammation are essential to confirm these findings.

## Conclusion

The findings of this systematic review and meta-analysis suggest that endodontic treatment may reduce serum levels of cardiovascular risk markers such as CRP, IL-6, and TNF-α in individuals with asymptomatic apical periodontitis. However, due to the low quality and high heterogeneity of the available evidence, these conclusions should be interpreted with caution.

## Supplementary Information

Below is the link to the electronic supplementary material.

Supplementary Material 1

Supplementary Material 2

Supplementary Material 3

Supplementary figure 1 (PNG 736 KB)

High Resolution Image (TIF 11.8 MB)Supplementary Fig. 1 Subgroup analysis of the effect of endodontic treatment on serum CRP levels. 1a. Baseline with external control group. 1b. Post-endodontic treatment with external control group

Supplementary figure 2 (PNG 660 KB)

High Resolution Image (TIF 11.8 MB)Supplementary Fig. 2 Subgroup analysis of the effect of endodontic treatment on serum IL-6 levels. 2a. Baseline with external control group. 2b. Post-endodontic treatment with external control group

Supplementary figure 3 (PNG 664 KB)

High Resolution Image (TIF 11.8 MB)Supplementary Fig. 3 Subgroup analysis of the effect of endodontic treatment on serum TNF-α levels. 3a. Baseline with external control group. 3b. Post-endodontic treatment with external control group

## Data Availability

All data included in this systematic review and meta-analysis were extracted from studies previously published in peer-reviewed journals or available in recognized scientific databases. No new data were generated by the authors. All sources of data are cited within the manuscript.
