# Supplementary material for: Does endodontic treatment modify serum inflammatory markers of cardiovascular risk in individuals with asymptomatic apical periodontitis? a systematic review and meta-analysis
Source: Clin Oral Investig. 2026 May 2;30(5):214. doi: 10.1007/s00784-026-06857-0 (PMC13135587; doi:10.1007/s00784-026-06857-0)
Supplement: Supplementary file 1 — Supplementary Material 1 [file 784_2026_6857_MOESM1_ESM.docx]

**Supplementary Information**

**Article title**: Does endodontic treatment modify serum inflammatory markers of cardiovascular risk in individuals with asymptomatic apical periodontitis? A systematic review and meta-analysis.

**Journal name:** Clinical Oral Investigations

**Author names:** Randerson Silva Araújo, Erika Bárbara Abreu Fonseca Thomaz, Juliana Balbinot Hilgert, Elma Izze da Silva Magalhães, Soraia de Fatima Carvalho Souza*

**Author Contributions:** Randerson Silva Araújo: Conceptualization; Methodology; Writing – Original Draft. Erika Bárbara Abreu Fonseca Thomaz: Conceptualization; Methodology; Formal Analysis; Writing –Review & Editing. Juliana Balbinot Hilgert: Writing – Review & Editing. Elma Izze da Silva Magalhães: Methodology; Formal Analysis. Soraia de Fátima Carvalho Souza: Conceptualization; Methodology; Writing – Original Draft; Writing – Review & Editing; Supervision.

*** Corresponding author**: Soraia de Fatima Carvalho Souza
Postgraduate Program in Dentistry, Federal University of Maranhão (UFMA), São Luís, MA, Brazil

*Email:* [endosoraia@gmail.com](mailto:endosoraia@gmail.com)

**Supplementary Table 1** Description of the search strategy conducted in the PubMed database and its respective results.

|  | Search Strategy | Results |
| --- | --- | --- |
| (((((((((((((((((((((((((((((((((((((((((((((((((((((((((((((((((((((((((((((((((((((((((((“Periapical Diseases”) OR “Disease, Periapical”) OR “Diseases, Periapical”) OR “Periapical Periodontitis”) OR “Periapical Disease”) OR “Periapical Tissue”) OR “Cyst, Radicular”) OR “Cysts, Radicular”) OR “Radicular Cysts”) OR “Periapical Cyst”) OR “Cyst, Periapical”) OR “Cysts, Periapical”) OR “Periapical Cysts”) OR “Periodontal Cyst, Apical”) OR “Apical Periodontal Cyst”) OR “Apical Periodontal Cysts”) OR “Cyst, Apical Periodontal”) OR “Cysts, Apical Periodontal”) OR “Periodontal Cysts, Apical”) OR “Radicular Cyst”) OR “Periodontitis, Acute Nonsuppurative”) OR “Acute Nonsuppurative Periodontitides”) OR “Acute Nonsuppurative Periodontitis”) OR “Periapical Granuloma”) OR “Granuloma, Periapical”) OR “Granulomas, Periapical”) OR “Periapical Granulomas”) OR “Periapical Periodontitis, Chronic Nonsuppurative”) OR “Periodontitis, Apical, Chronic Nonsuppurative”) OR “Dental Granulomas”) OR “Granulomas, Dental”) OR “Dental Granuloma”) OR “Granuloma, Dental”) OR “Nonsuppurative Periodontitides, Acute”) OR “Nonsuppurative Periodontitis, Acute”) OR “Periodontitides, Acute Nonsuppurative”) OR “Periapical Periodontitides”) OR “Periodontitides, Periapical”) OR “Periodontitis, Periapical”) OR “Periodontitis, Apical”) OR “Apical Periodontitides”) OR “Apical Periodontitis”) OR “Periodontitides, Apical”) OR “Periapical Abscess”) OR “Periodontitis, Apical, Suppurative”) OR “Abscess, Periapical”) OR “Abscesses, Periapical”) OR “Periapical Abscesses”) OR “Periapical Periodontitis, Suppurative”) OR “Periapical Periodontitides, Suppurative”) OR “Periodontitides, Suppurative Periapical”) OR “Periodontitis, Suppurative Periapical”) OR “Suppurative Periapical Periodontitides”) OR “Suppurative Periapical Periodontitis”) OR “Alveolar Abscess, Apical”) OR “Abscess, Apical Alveolar”) OR “Abscesses, Apical Alveolar”) OR “Alveolar Abscesses, Apical”) OR “Apical Alveolar Abscess”) OR “Apical Alveolar Abscesses”) OR “Dentoalveolar Abscess, Apical”) OR “Abscess, Apical Dentoalveolar”) OR “Abscesses, Apical Dentoalveolar”) OR “Apical Dentoalveolar Abscess”) OR “Apical Dentoalveolar Abscesses”) OR “Dentoalveolar Abscesses, Apical”) OR “Periapical Tissues”) OR “Tissue, Periapical”) OR “Tissues, Periapical”) OR “Periodontium, Apical”) OR “Apical Periodontium”) OR “Apical Periodontiums”) OR “Periodontiums, Apical”) OR “Tooth, Nonvital”) OR “Nonvital Tooth”) OR “Tooth, Devitalized”) OR “Devitalized Tooth”) OR “Tooth, Pulpless”) OR “Pulpless Tooth”) OR “Teeth, Pulpless”) OR “Teeth, Devitalized”) OR “Devitalized Teeth”) OR “Teeth, Nonvital”) OR “Nonvital Teeth”) OR “Lesions of Endodontic Origin”) OR “Periapical Lesions”) OR “Apical Lesions”) OR “Periradicular Lesions”)) AND ((((((((((((((((((“Root Canal Therapy”) OR “Canal Therapies, Root”) OR “Canal Therapy, Root”) OR “Root Canal Therapies”) OR “Therapies, Root Canal”) OR “Therapy, Root Canal”) OR Endodontics) OR Endodontology) OR Apicoectomy) OR Apicoectomies) OR "Root Canal Preparation") OR "Preparation, Root Canal") OR "Root Canal Preparations") OR "Access Cavity Preparation") OR "Access Cavity Preparations") OR "Preparation, Access Cavity") OR "Endodontics Access Cavity Preparation") OR "Root Canal Treatment")) AND (((((((((((((((((((((((((((((((((((((((((((((((((((((((((((((((((((((((((((((((((((((((((((((((((((((((“Inflammation Mediators”) OR “Mediators, Inflammation”) OR “Mediators of Inflammation”) OR Cytokines) OR Cytokine) OR Chemokines) OR “Chemotactic Cytokine”) OR “Cytokine, Chemotactic”) OR Intercrines) OR “Chemotactic Cytokines”) OR “Cytokines, Chemotactic”) OR Intercrine) OR Chemokine) OR Interleukins) OR Interleukin) OR “C-Reactive Protein”) OR “C Reactive Protein”) OR hsCRP) OR “High Sensitivity C-Reactive Protein”) OR “High Sensitivity C Reactive Protein”) OR hs-CRP) OR “Tumor Necrosis Factor-alpha”) OR “Tumor Necrosis Factor alpha”) OR “Cachectin”) OR “Cachectin-Tumor Necrosis Factor”) OR “Cachectin Tumor Necrosis Factor”) OR “Tumor Necrosis Factor Ligand Superfamily Member 2”) OR “Tumor Necrosis Factor”) OR “TNF Superfamily, Member 2”) OR TNFalpha) OR “TNF-alpha”) OR “Vascular Cell Adhesion Molecule-1”) OR “VCAM 1”) OR “Vascular Cell Adhesion Molecule 1”) OR “Inducible Cell Adhesion Molecule 110”) OR “INCAM-110”) OR “CD106 Antigens”) OR “CD106 Antigen”) OR “Antigen, CD106”) OR “Antigens, CD106”) OR “Vascular Cell Adhesion Molecule”) OR “VCAM-1”) OR “Cell Adhesion Molecules”) OR “Adhesion Molecules, Cell”) OR “Molecules, Cell Adhesion”) OR “Cell Adhesion Molecule”) OR “Adhesion Molecule, Cell”) OR “Molecule, Cell Adhesion”) OR “Leukocyte Adhesion Molecules”) OR “Adhesion Molecules, Leukocyte”) OR “Molecules, Leukocyte Adhesion”) OR “Leukocyte Adhesion Molecule”) OR “Adhesion Molecule, Leukocyte”) OR “Molecule, Leukocyte Adhesion”) OR “Saccharide-Mediated Cell Adhesion Molecules”) OR “Saccharide Mediated Cell Adhesion Molecules”) OR “Intercellular Adhesion Molecules”) OR “Adhesion Molecules, Intercellular”) OR “Molecules, Intercellular Adhesion”) OR “Intercellular Adhesion Molecule”) OR “Adhesion Molecule, Intercellular”) OR “Antigens, Surface”) OR Glycoproteins) OR “Membrane Glycoproteins”) OR “Surface Antigens”) OR “Cell Surface Antigen”) OR “Antigen, Cell Surface”) OR “Surface Markers, Immunological”) OR “Immunologic Surface Markers”) OR “Markers, Immunologic Surface”) OR “Surface Markers, Immunologic”) OR “Immunological Surface Markers”) OR “Surface Antigen”) OR “Antigen, Surface”) OR “Cell Surface Antigens”) OR Glycoprotein) OR “Proteins, N-Glycosylated”) OR “O-Glycosylated Proteins”) OR “Proteins, O-Glycosylated”) OR “Glycosylated Proteins”) OR “Proteins, Glycosylated”) OR “Glycosylated Protein”) OR “Protein, Glycosylated”) OR “C-Glycosylated Proteins”) OR “Proteins, C-Glycosylated”) OR Neoglycoproteins) OR “Glycoprotein, Membrane”) OR “Glycoprotein, Surface”) OR “Surface Glycoproteins”) OR “Cell Surface Glycoprotein”) OR “Glycoprotein, Cell Surface”) OR “Cell Surface Glycoproteins”) OR “Glycoproteins, Cell Surface”) OR “Surface Glycoproteins, Cell”) OR “ICAM 1”) OR “Intercellular Adhesion Molecule 1”) OR “ICAM-1”) OR “CD54 Antigens”) OR “Antigen, CD54”) OR “Antigens, CD54”) OR “ICAM-1 Protein, Human”) OR “Intercellular Adhesion Molecule 1p, Human”) OR “Acute Phase Proteins”) | | 3444 |

**Supplementary Table 2** Description of the search strategy conducted in the Web of Science database and its respective results.

| Search Strategy | Results |
| --- | --- |
| #1 AND #2 | 471 |
| #1 AND #2 AND #3 | 11 |
| ALL= (“Root Canal Therapy” OR “Canal Therapies, Root” OR “Canal Therapy, Root” OR “Root Canal Therapies” OR “Therapies, Root Canal” OR “Therapy, Root Canal”) | 1.213 |
| ALL=(“Inflammation Mediators” OR “Mediators, Inflammation” OR “Mediators of Inflammation” OR Cytokines OR Cytokine OR Chemokines OR “Chemotactic Cytokine” OR “Cytokine, Chemotactic” OR Intercrines OR “Chemotactic Cytokines” OR “Cytokines, Chemotactic” OR Intercrine OR Chemokine OR Interleukins OR Interleukin OR “C-Reactive Protein” OR “C Reactive Protein” OR hsCRP OR “High Sensitivity C-Reactive Protein” OR “High Sensitivity C Reactive Protein” OR hs-CRP OR “Tumor Necrosis Factor-alpha” OR “Tumor Necrosis Factor alpha” OR “Cachectin” OR “Cachectin-Tumor Necrosis Factor” OR “Cachectin Tumor Necrosis Factor” OR “Tumor Necrosis Factor Ligand Superfamily Member 2” OR “Tumor Necrosis Factor” OR “TNF Superfamily, Member 2” OR TNFalpha OR TNF-alpha) | 970.144 |
| ALL=(“Periapical Diseases” OR “Disease, Periapical” OR “Diseases, Periapical” OR “Periapical Periodontitis” OR “Periapical Disease” OR “Periapical Periodontitides” OR “Periodontitides, Periapical” OR “Periodontitis, Periapical” OR “Periodontitis, Apical” OR “Apical Periodontitides” OR “Apical Periodontitis” OR “Periodontitides, Apical” OR “Periapical Granuloma” OR “Granuloma, Periapical” OR “Granulomas, Periapical” OR “Periapical Granulomas” OR “Periapical Periodontitis, Chronic Nonsuppurative” OR “Periodontitis, Apical, Chronic Nonsuppurative” OR “Dental Granulomas” OR “Granulomas, Dental” OR “Dental Granuloma” OR “Granuloma, Dental”) | 3.860 |

| Search Strategy  **Supplementary Table 3** Description of the search strategy conducted in the Scopus database and its respective results. | Results |
| --- | --- |
| ( ( ( ( ( ( ( ( ( ( ( ( ( ( ( ( ( ( ( ( ( ( ( ( ( ( ( ( ( ( ( ( ( ( ( ( ( ( ( ( ( ( ( ( ( ( ( ( ( ( ( ( ( ( ( ( "Periapical Diseases" [all AND fields] OR "Disease, Periapical" [all AND fields] ) OR "Diseases, Periapical" [all AND fields] ) OR "Periapical Periodontitis" [all AND fields] ) OR "Periapical Disease" [all AND fields] ) OR "Periapical Tissue" [all AND fields] ) OR "Cyst, Radicular" [all AND fields] ) OR "Cysts, Radicular" [all AND fields] ) OR "Radicular Cysts" [all AND fields] ) OR "Periapical Cyst" [all AND fields] ) OR "Cyst, Periapical" [all AND fields] ) OR "Cysts, Periapical" [all AND fields] ) OR "Periapical Cysts" [all AND fields] ) OR "Periodontal Cyst, Apical" [all AND fields] ) OR "Apical Periodontal Cyst" [all AND fields] ) OR "Apical Periodontal Cysts" [all AND fields] ) OR "Cyst, Apical Periodontal" [all AND fields] ) OR "Radicular Cyst" [all AND fields] ) OR "Periapical Granuloma" [all AND fields] ) OR "Granuloma, Periapical" [all AND fields] ) OR "Granulomas, Periapical" [all AND fields] ) OR "Periapical Granulomas" [all AND fields] ) OR "Dental Granulomas" [all AND fields] ) OR "Granulomas, Dental" [all AND fields] ) OR "Dental Granuloma" [all AND fields] ) OR "Granuloma, Dental" [all AND fields] ) OR "Periapical Periodontitides" [all AND fields] ) OR "Periodontitis, Periapical" [all AND fields] ) OR "Periodontitis, Apical" [all AND fields] ) OR "Apical Periodontitis" [all AND fields] ) OR "Periapical Abscess" [all AND fields] ) OR "Abscess, Periapical" [all AND fields] ) OR "Abscesses, Periapical" [all AND fields] ) OR "Periapical Abscesses" [all AND fields] ) OR "Suppurative Periapical Periodontitis" [all AND fields] ) OR "Alveolar Abscess, Apical" [all AND fields] ) OR "Apical Alveolar Abscess" [all AND fields] ) OR "Periapical Tissues" [all AND fields] ) OR "Tissue, Periapical" [all AND fields] ) OR "Tissues, Periapical" [all AND fields] ) OR "Periodontium, Apical" [all AND fields] ) OR "Apical Periodontium" [all AND fields] ) OR "Tooth, Nonvital" [all AND fields] ) OR "Nonvital Tooth" [all AND fields] ) OR "Devitalized Tooth" [all AND fields] ) OR "Pulpless Tooth" [all AND fields] ) OR "Teeth, Pulpless" [all AND fields] ) OR "Pulpless Teeth" [all AND fields] ) OR "Teeth, Devitalized" [all AND fields] ) OR "Devitalized Teeth" [all AND fields] ) OR "Teeth, Nonvital" [all AND fields] ) OR "Nonvital Teeth" [all AND fields] ) OR "Lesions of Endodontic Origin" [all AND fields] ) OR "Periapical Lesions" [all AND fields] ) OR "Apical Lesions" [all AND fields] ) OR "Periradicular Lesions" [all AND fields] ) AND ( ( ( ( ( ( ( ( ( ( ( ( ( ( ( ( "Root Canal Therapy" [all AND fields] OR "Canal Therapies, Root" [all AND fields] ) OR "Canal Therapy, Root" [all AND fields] ) OR "Root Canal Therapies" [all AND fields] ) OR "Therapies, Root Canal" [all AND fields] ) OR "Therapy, Root Canal" [all AND fields] ) OR ( "endodontics" [mesh AND terms] OR "endodontics" [all AND fields] ) ) OR ( "endodontics" [mesh AND terms] OR "endodontics" [all AND fields] OR "endodontology" [all AND fields] ) ) OR ( "apicoectomy" [mesh AND terms] OR "apicoectomy" [all AND fields] ) ) OR ( "apicoectomy" [mesh AND terms] OR "apicoectomy" [all AND fields] OR "apicoectomies" [all AND fields] ) ) OR "Root Canal Preparation" [all AND fields] ) OR "Preparation, Root Canal" [all AND fields] ) OR "Root Canal Preparations" [all AND fields] ) OR "Access Cavity Preparation" [all AND fields] ) OR "Access Cavity Preparations" [all AND fields] ) OR "Preparation, Access Cavity" [all AND fields] ) OR "Root Canal Treatment" [all AND fields] ) ) AND ( ( ( ( ( ( ( ( ( ( ( ( ( ( ( ( ( ( ( ( ( ( ( ( ( ( ( ( ( ( ( ( ( ( ( ( ( ( ( ( ( ( ( ( ( ( ( ( ( ( ( ( ( ( ( ( ( ( ( ( ( ( ( ( ( ( ( ( ( ( ( ( ( ( ( ( ( ( ( ( ( ( ( ( ( ( ( ( ( ( ( ( ( ( ( ( ( ( ( ( ( ( ( ( ( ( ( ( ( ( ( "Inflammation Mediators" [all AND fields] OR "Mediators, Inflammation" [all AND fields] ) OR "Mediators of Inflammation" [all AND fields] ) OR ( "cytokines" [mesh AND terms] OR "cytokines" [all AND fields] ) ) OR ( "cytokines" [mesh AND terms] OR "cytokines" [all AND fields] OR "cytokine" [all AND fields] ) ) OR ( "chemokines" [mesh AND terms] OR "chemokines" [all AND fields] ) ) OR "Chemotactic Cytokine" [all AND fields] ) OR "Cytokine, Chemotactic" [all AND fields] ) OR ( "chemokines" [mesh AND terms] OR "chemokines" [all AND fields] OR "intercrines" [all AND fields] ) ) OR "Chemotactic Cytokines" [all AND fields] ) OR "Cytokines, Chemotactic" [all AND fields] ) OR ( "chemokines" [mesh AND terms] OR "chemokines" [all AND fields] OR "intercrine" [all AND fields] ) ) OR ( "chemokines" [mesh AND terms] OR "chemokines" [all AND fields] OR "chemokine" [all AND fields] ) ) OR ( "interleukins" [mesh AND terms] OR "interleukins" [all AND fields] ) ) OR ( "interleukins" [mesh AND terms] OR "interleukins" [all AND fields] OR "interleukin" [all AND fields] ) ) OR "C-Reactive Protein" [all AND fields] ) OR "C Reactive Protein" [all AND fields] ) OR ( "c-reactive protein" [mesh AND terms] OR ( "c-reactive" [all AND fields] AND "protein" [all AND fields] ) OR "c-reactive protein" [all AND fields] OR "hscrp" [all AND fields] ) ) OR "High Sensitivity C-Reactive Protein" [all AND fields] ) OR "High Sensitivity C Reactive Protein" [all AND fields] ) OR ( "c-reactive protein" [mesh AND terms] OR ( "c-reactive" [all AND fields] AND "protein" [all AND fields] ) OR "c-reactive protein" [all AND fields] OR ( "hs" [all AND fields] AND "crp" [all AND fields] ) OR "hs crp" [all AND fields] ) ) OR "Tumor Necrosis Factor-alpha" [all AND fields] ) OR "Tumor Necrosis Factor alpha" [all AND fields] ) OR "Cachectin" [all AND fields] ) OR "Cachectin-Tumor Necrosis Factor" [all AND fields] ) OR "Cachectin Tumor Necrosis Factor" [all AND fields] ) OR "Tumor Necrosis Factor Ligand Superfamily Member 2" [all AND fields] ) OR "Tumor Necrosis Factor" [all AND fields] ) OR "TNF Superfamily, Member 2" [all AND fields] ) OR ( "tumor necrosis factor-alpha" [mesh AND terms] OR ( "tumor" [all AND fields] AND "necrosis" [all AND fields] AND "factor-alpha" [all AND fields] ) OR "tumor necrosis factor-alpha" [all AND fields] OR "tnfalpha" [all AND fields] ) ) OR ( "tumor necrosis factor-alpha" [mesh AND terms] OR ( "tumor" [all AND fields] AND "necrosis" [all AND fields] AND "factor-alpha" [all AND fields] ) OR "tumor necrosis factor-alpha" [all AND fields] OR ( "tnf" [all AND fields] AND "alpha" [all AND fields] ) OR "tnf alpha" [all AND fields] ) ) OR "Vascular Cell Adhesion Molecule-1" [all AND fields] ) OR "VCAM 1" [all AND fields] ) OR "Vascular Cell Adhesion Molecule 1" [all AND fields] ) OR "Inducible Cell Adhesion Molecule 110" [all AND fields] ) OR "INCAM-110" [all AND fields] ) OR "CD106 Antigens" [all AND fields] ) OR "CD106 Antigen" [all AND fields] ) OR "Antigen, CD106" [all AND fields] ) OR "Antigens, CD106" [all AND fields] ) OR "Vascular Cell Adhesion Molecule" [all AND fields] ) OR "VCAM-1" [all AND fields] ) OR "Cell Adhesion Molecules" [all AND fields] ) OR "Adhesion Molecules, Cell" [all AND fields] ) OR "Molecules, Cell Adhesion" [all AND fields] ) OR "Cell Adhesion Molecule" [all AND fields] ) OR "Adhesion Molecule, Cell" [all AND fields] ) OR "Molecule, Cell Adhesion" [all AND fields] ) OR "Leukocyte Adhesion Molecules" [all AND fields] ) OR "Adhesion Molecules, Leukocyte" [all AND fields] ) OR "Molecules, Leukocyte Adhesion" [all AND fields] ) OR "Leukocyte Adhesion Molecule" [all AND fields] ) OR "Adhesion Molecule, Leukocyte" [all AND fields] ) OR "Intercellular Adhesion Molecules" [all AND fields] ) OR "Adhesion Molecules, Intercellular" [all AND fields] ) OR "Molecules, Intercellular Adhesion" [all AND fields] ) OR "Intercellular Adhesion Molecule" [all AND fields] ) OR "Adhesion Molecule, Intercellular" [all AND fields] ) OR "Molecule, Intercellular Adhesion" [all AND fields] ) OR "Antigens, Surface" [all AND fields] ) OR ( "glycoproteins" [mesh AND terms] OR "glycoproteins" [all AND fields] ) ) OR "Membrane Glycoproteins" [all AND fields] ) OR "Surface Antigens" [all AND fields] ) OR "Cell Surface Antigen" [all AND fields] ) OR "Antigen, Cell Surface" [all AND fields] ) OR "Surface Antigen, Cell" [all AND fields] ) OR "Surface Markers, Immunological" [all AND fields] ) OR "Immunologic Surface Markers" [all AND fields] ) OR "Surface Markers, Immunologic" [all AND fields] ) OR "Immunological Surface Markers" [all AND fields] ) OR "Surface Antigen" [all AND fields] ) OR "Antigen, Surface" [all AND fields] ) OR "Cell Surface Antigens" [all AND fields] ) OR "Antigens, Cell Surface" [all AND fields] ) OR "Surface Antigens, Cell" [all AND fields] ) OR ( "glycoproteins" [mesh AND terms] OR "glycoproteins" [all AND fields] OR "glycoprotein" [all AND fields] ) ) OR "N-Glycosylated Proteins" [all AND fields] ) OR "Proteins, N-Glycosylated" [all AND fields] ) OR "O-Glycosylated Proteins" [all AND fields] ) OR "Proteins, O-Glycosylated" [all AND fields] ) OR "Glycosylated Proteins" [all AND fields] ) OR "Proteins, Glycosylated" [all AND fields] ) OR "Glycosylated Protein" [all AND fields] ) OR "Protein, Glycosylated" [all AND fields] ) OR "C-Glycosylated Proteins" [all AND fields] ) OR ( "glycoproteins" [mesh AND terms] OR "glycoproteins" [all AND fields] OR "neoglycoproteins" [all AND fields] ) ) OR "Glycoproteins, Membrane" [all AND fields] ) OR "Membrane Glycoprotein" [all AND fields] ) OR "Glycoprotein, Membrane" [all AND fields] ) OR "Surface Glycoprotein" [all AND fields] ) OR "Glycoprotein, Surface" [all AND fields] ) OR "Surface Glycoproteins" [all AND fields] ) OR "Glycoproteins, Surface" [all AND fields] ) OR "Cell Surface Glycoprotein" [all AND fields] ) OR "Glycoprotein, Cell Surface" [all AND fields] ) OR "Surface Glycoprotein, Cell" [all AND fields] ) OR "Cell Surface Glycoproteins" [all AND fields] ) OR "Glycoproteins, Cell Surface" [all AND fields] ) OR "Surface Glycoproteins, Cell" [all AND fields] ) OR "Intercellular Adhesion Molecule-1" [all AND fields] ) OR "ICAM 1" [all AND fields] ) OR "Intercellular Adhesion Molecule 1" [all AND fields] ) OR "ICAM-1" [all AND fields] ) OR "CD54 Antigens" [all AND fields] ) OR "CD54 Antigen" [all AND fields] ) OR "Antigen, CD54" [all AND fields] ) OR "Antigens, CD54" [all AND fields] ) OR ( "immunoproteins" [mesh AND terms] OR "immunoproteins" [all AND fields] ) ) OR endopeptidades[all AND fields] ) OR "Acute Phase Proteins" [all AND fields] ) OR ( "blood" [subheading] OR "blood" [all AND fields] OR "blood" [mesh AND terms] ) ) OR ( "serum" [mesh AND terms] OR "serum" [all AND fields] ) ) | 103 |

**Supplementary Table 4** Description of the search strategy conducted in the Embase database and its respective results.

| Search Strategy | Results |
| --- | --- |
| ('periapical diseases'/exp OR 'periapical diseases' OR 'disease, periapical' OR 'diseases, periapical' OR 'periapical periodontitis'/exp OR 'periapical periodontitis' OR 'periapical disease'/exp OR 'periapical disease' OR 'periapical tissue'/exp OR 'periapical tissue' OR 'cyst, radicular' OR 'cysts, radicular' OR 'radicular cysts' OR 'periapical cyst'/exp OR 'periapical cyst' OR 'cyst, periapical' OR 'cysts, periapical' OR 'periapical cysts' OR 'periodontal cyst, apical' OR 'apical periodontal cyst'/exp OR 'apical periodontal cyst' OR 'apical periodontal cysts' OR 'cyst, apical periodontal' OR 'cysts, apical periodontal' OR 'periodontal cysts, apical' OR 'radicular cyst'/exp OR 'radicular cyst' OR 'periodontitis, acute nonsuppurative' OR 'acute nonsuppurative periodontitides' OR 'acute nonsuppurative periodontitis' OR 'periapical granuloma'/exp OR 'periapical granuloma' OR 'granuloma, periapical' OR 'granulomas, periapical' OR 'periapical granulomas' OR 'periapical periodontitis, chronic nonsuppurative' OR 'periodontitis, apical, chronic nonsuppurative' OR 'dental granulomas' OR 'granulomas, dental' OR 'dental granuloma'/exp OR 'dental granuloma' OR 'granuloma, dental' OR 'nonsuppurative periodontitides, acute' OR 'nonsuppurative periodontitis, acute' OR 'periodontitides, acute nonsuppurative' OR 'periapical periodontitides' OR 'periodontitides, periapical' OR 'periodontitis, periapical' OR 'periodontitis, apical' OR 'apical periodontitides' OR 'apical periodontitis'/exp OR 'apical periodontitis' OR 'periodontitides, apical' OR 'periapical abscess'/exp OR 'periapical abscess' OR 'periodontitis, apical, suppurative' OR 'abscess, periapical' OR 'abscesses, periapical' OR 'periapical abscesses'/exp OR 'periapical abscesses' OR 'periapical periodontitis, suppurative' OR 'periapical periodontitides, suppurative' OR 'periodontitides, suppurative periapical' OR 'periodontitis, suppurative periapical' OR 'suppurative periapical periodontitides' OR 'suppurative periapical periodontitis'/exp OR 'suppurative periapical periodontitis' OR 'alveolar abscess, apical' OR 'abscess, apical alveolar' OR 'abscesses, apical alveolar' OR 'alveolar abscesses, apical' OR 'apical alveolar abscess' OR 'apical alveolar abscesses' OR 'dentoalveolar abscess, apical' OR 'abscess, apical dentoalveolar' OR 'abscesses, apical dentoalveolar' OR 'apical dentoalveolar abscess' OR 'apical dentoalveolar abscesses' OR 'dentoalveolar abscesses, apical' OR 'periapical tissues' OR 'tissue, periapical' OR 'tissues, periapical' OR 'periodontium, apical' OR 'apical periodontium' OR 'apical periodontiums' OR 'periodontiums, apical' OR 'tooth, nonvital'/exp OR 'tooth, nonvital' OR 'nonvital tooth'/exp OR 'nonvital tooth' OR 'tooth, devitalized' OR 'devitalized tooth' OR 'tooth, pulpless' OR 'pulpless tooth' OR 'teeth, pulpless' OR 'teeth, devitalized' OR 'devitalized teeth' OR 'teeth, nonvital' OR 'nonvital teeth' OR 'lesions of endodontic origin' OR 'periapical lesions' OR 'apical lesions' OR 'periradicular lesions') AND ('root canal therapy'/exp OR 'root canal therapy' OR 'canal therapies, root' OR 'canal therapy, root' OR 'root canal therapies' OR 'therapies, root canal' OR 'therapy, root canal' OR 'endodontics'/exp OR endodontics OR endodontology OR 'apicoectomy'/exp OR apicoectomy OR apicoectomies OR 'root canal preparation'/exp OR 'root canal preparation' OR 'preparation, root canal' OR 'root canal preparations' OR 'access cavity preparation' OR 'access cavity preparations' OR 'preparation, access cavity' OR 'endodontics access cavity preparation' OR 'root canal treatment'/exp OR 'root canal treatment') AND ('inflammation mediators'/exp OR 'inflammation mediators' OR 'mediators, inflammation' OR 'mediators of inflammation' OR 'cytokines'/exp OR cytokines OR 'cytokine'/exp OR cytokine OR 'chemokines'/exp OR chemokines OR 'chemotactic cytokine'/exp OR 'chemotactic cytokine' OR 'cytokine, chemotactic' OR intercrines OR 'chemotactic cytokines' OR 'cytokines, chemotactic' OR intercrine OR 'chemokine'/exp OR chemokine OR 'interleukins'/exp OR interleukins OR 'interleukin'/exp OR interleukin OR 'c-reactive protein'/exp OR 'c-reactive protein' OR 'c reactive protein'/exp OR 'c reactive protein' OR hscrp OR 'high sensitivity c-reactive protein' OR 'high sensitivity c reactive protein'/exp OR 'high sensitivity c reactive protein' OR 'hs crp' OR 'tumor necrosis factor-alpha'/exp OR 'tumor necrosis factor-alpha' OR 'tumor necrosis factor alpha'/exp OR 'tumor necrosis factor alpha' OR 'cachectin'/exp OR 'cachectin' OR 'cachectin-tumor necrosis factor' OR 'cachectin tumor necrosis factor' OR 'tumor necrosis factor ligand superfamily member 2' OR 'tumor necrosis factor'/exp OR 'tumor necrosis factor' OR 'tnf superfamily, member 2' OR tnfalpha OR 'tnf-alpha'/exp OR 'tnf-alpha' OR 'vascular cell adhesion molecule-1'/exp OR 'vascular cell adhesion molecule-1' OR 'vcam 1'/exp OR 'vcam 1' OR 'vascular cell adhesion molecule 1'/exp OR 'vascular cell adhesion molecule 1' OR 'inducible cell adhesion molecule 110' OR 'incam-110' OR 'cd106 antigens' OR 'cd106 antigen'/exp OR 'cd106 antigen' OR 'antigen, cd106' OR 'antigens, cd106' OR 'vascular cell adhesion molecule'/exp OR 'vascular cell adhesion molecule' OR 'vcam-1'/exp OR 'vcam-1' OR 'cell adhesion molecules'/exp OR 'cell adhesion molecules' OR 'adhesion molecules, cell' OR 'molecules, cell adhesion' OR 'cell adhesion molecule'/exp OR 'cell adhesion molecule' OR 'adhesion molecule, cell' OR 'molecule, cell adhesion' OR 'leukocyte adhesion molecules' OR 'adhesion molecules, leukocyte' OR 'molecules, leukocyte adhesion' OR 'leukocyte adhesion molecule' OR 'adhesion molecule, leukocyte' OR 'molecule, leukocyte adhesion' OR 'saccharide-mediated cell adhesion molecules' OR 'saccharide mediated cell adhesion molecules' OR 'intercellular adhesion molecules' OR 'adhesion molecules, intercellular' OR 'molecules, intercellular adhesion' OR 'intercellular adhesion molecule'/exp OR 'intercellular adhesion molecule' OR 'adhesion molecule, intercellu  lar' OR 'antigens, surface'/exp OR 'antigens, surface' OR 'glycoproteins'/exp OR glycoproteins OR 'membrane glycoproteins'/exp OR 'membrane glycoproteins' OR 'surface antigens' OR 'cell surface antigen'/exp OR 'cell surface antigen' OR 'antigen, cell surface' OR 'surface markers, immunological' OR 'immunologic surface markers' OR 'markers, immunologic surface' OR 'surface markers, immunologic' OR 'immunological surface markers' OR 'surface antigen'/exp OR 'surface antigen' OR 'antigen, surface' OR 'cell surface antigens' OR 'glycoprotein'/exp OR glycoprotein OR 'proteins, n-glycosylated' OR 'o-glycosylated proteins' OR 'proteins, o-glycosylated' OR 'glycosylated proteins' OR 'proteins, glycosylated' OR 'glycosylated protein'/exp OR 'glycosylated protein' OR 'protein, glycosylated' OR 'c-glycosylated proteins' OR 'proteins, c-glycosylated' OR neoglycoproteins OR 'glycoprotein, membrane' OR 'glycoprotein, surface' OR 'surface glycoproteins' OR 'cell surface glycoprotein'/exp OR 'cell surface glycoprotein' OR 'glycoprotein, cell surface'/exp OR 'glycoprotein, cell surface' OR 'cell surface glycoproteins' OR 'glycoproteins, cell surface' OR 'surface glycoproteins, cell' OR 'icam 1'/exp OR 'icam 1' OR 'intercellular adhesion molecule 1'/exp OR 'intercellular adhesion molecule 1' OR 'icam-1'/exp OR 'icam-1' OR 'cd54 antigens' OR 'antigen, cd54' OR 'antigens, cd54' OR 'icam-1 protein, human' OR 'intercellular adhesion molecule 1p, human' OR 'acute phase proteins'/exp OR 'acute phase proteins') | 767 |

**Supplementary Table 5** Description of the search strategy conducted in the VHL database (Medline, IBECS, BBO, LILACS) and its respective results.

| Number | Search Strategy | Results |
| --- | --- | --- |
| **#1** | (Periapical Diseases”) OR ("Periapical Periodontitis") OR (“Periapical Cyst”)OR (“Periapical Granuloma”) AND (“Root Canal Therapy”) OR (“Root Canal Therapies”) OR ("Root Canal Preparation") AND(“Inflammation Mediators”) OR (Cytokines) | 387 |

| Database | Search Strategy | Results |
| --- | --- | --- |
| Google Scholar | ("periapical periodontitis" OR "apical periodontitis") AND (cytokines OR "inflammatory mediators" OR "C reactive protein") AND (“root canal therapy”) | 965 |
| CAPES Theses and Dissertations Catalog | “periodontite apical” AND “marcadores inflamatórios) | 5 |
| PROQUEST | ("periapical periodontitis") AND (cytokines OR "inflammatory mediators" OR "C reactive protein") AND (“root canal therapy”) | 153 |
| Brazilian Digital Library of Theses and Dissertations (BDTD)  Manual search in reference lists | "(Todos os campos:periodontite apical assintomática E Todos os campos:marcadores inflamatórios)" | 0  2 |

**Supplementary Table 6** Description of the search strategy conducted in the gray literature databases and its respective results.
