# Supplementary figures and images for: Does endodontic treatment modify serum inflammatory markers of cardiovascular risk in individuals with asymptomatic apical periodontitis? a systematic review and meta-analysis
Source: Clin Oral Investig. 2026 May 2;30(5):214. doi: 10.1007/s00784-026-06857-0 (PMC13135587; doi:10.1007/s00784-026-06857-0)

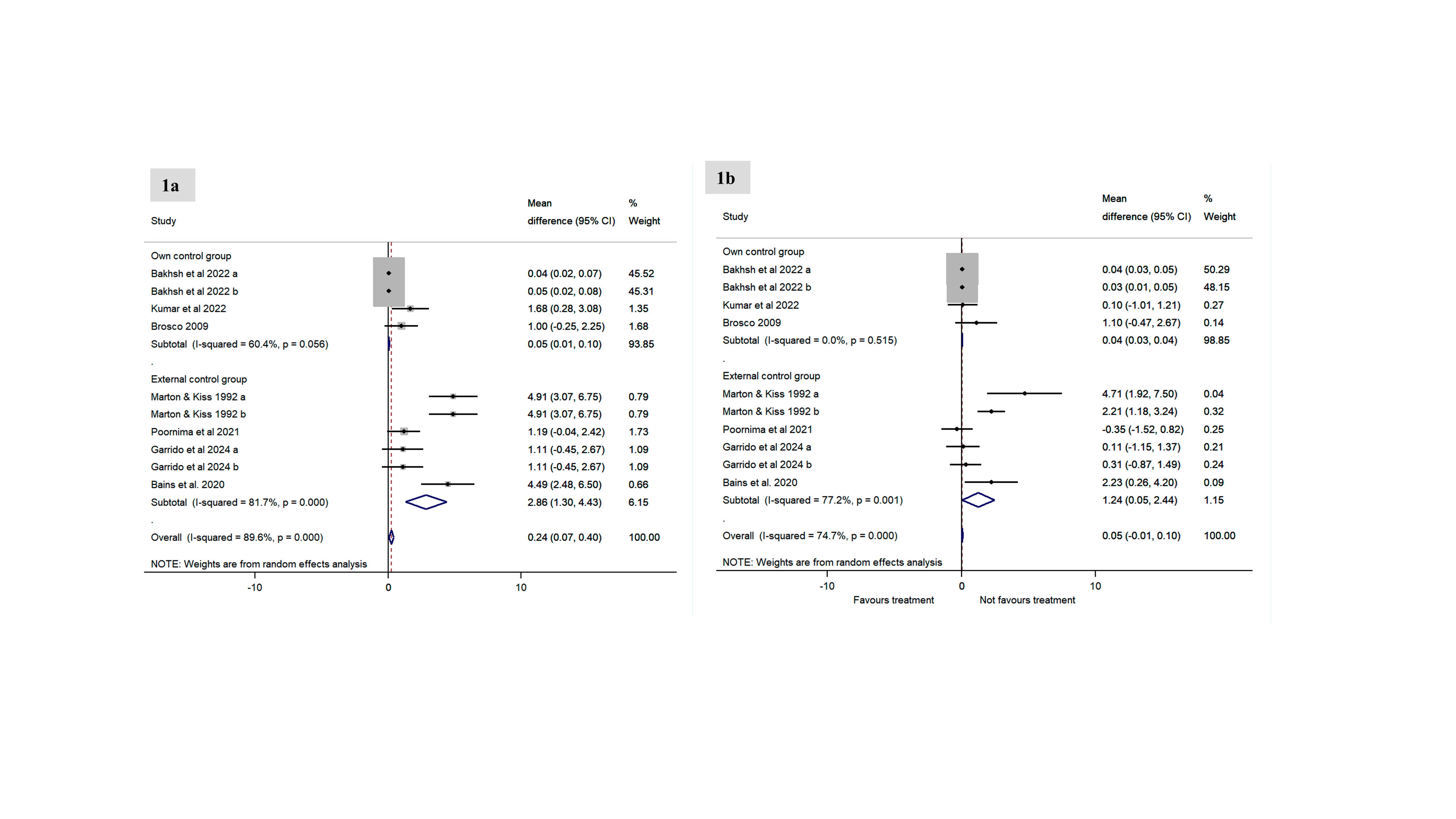

Supplement: Supplementary file 4 — (PNG 736 KB) [file 784_2026_6857_Fig5_ESM.png]

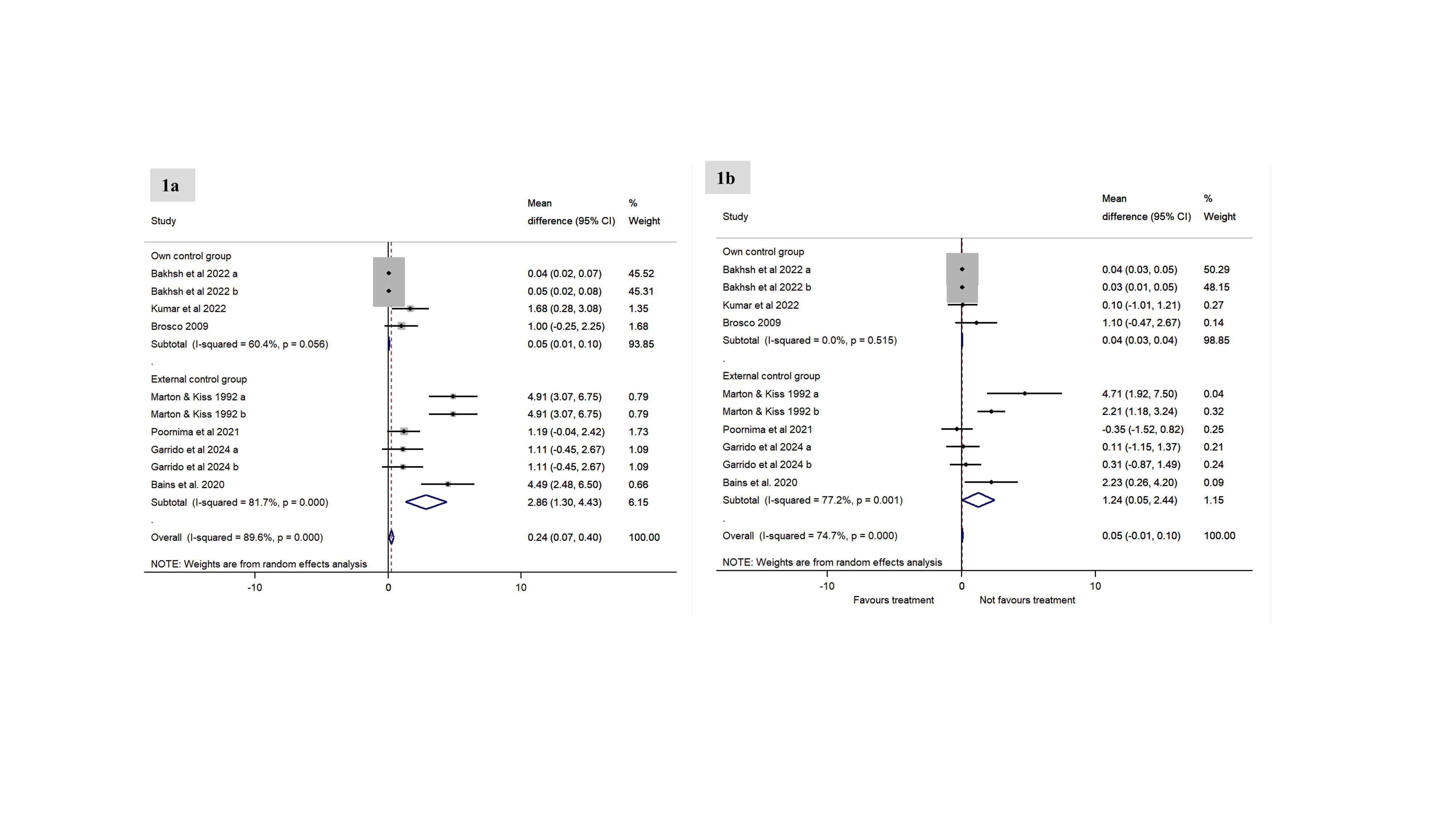

Supplement: Supplementary file 5 — High Resolution Image (TIF 11.8 MB)Supplementary Fig. 1 Subgroup analysis of the effect of endodontic treatment on serum CRP levels. 1a. Baseline with external control group. 1b. Post-endodontic treatment with external control group [file 784_2026_6857_MOESM4_ESM.tif]

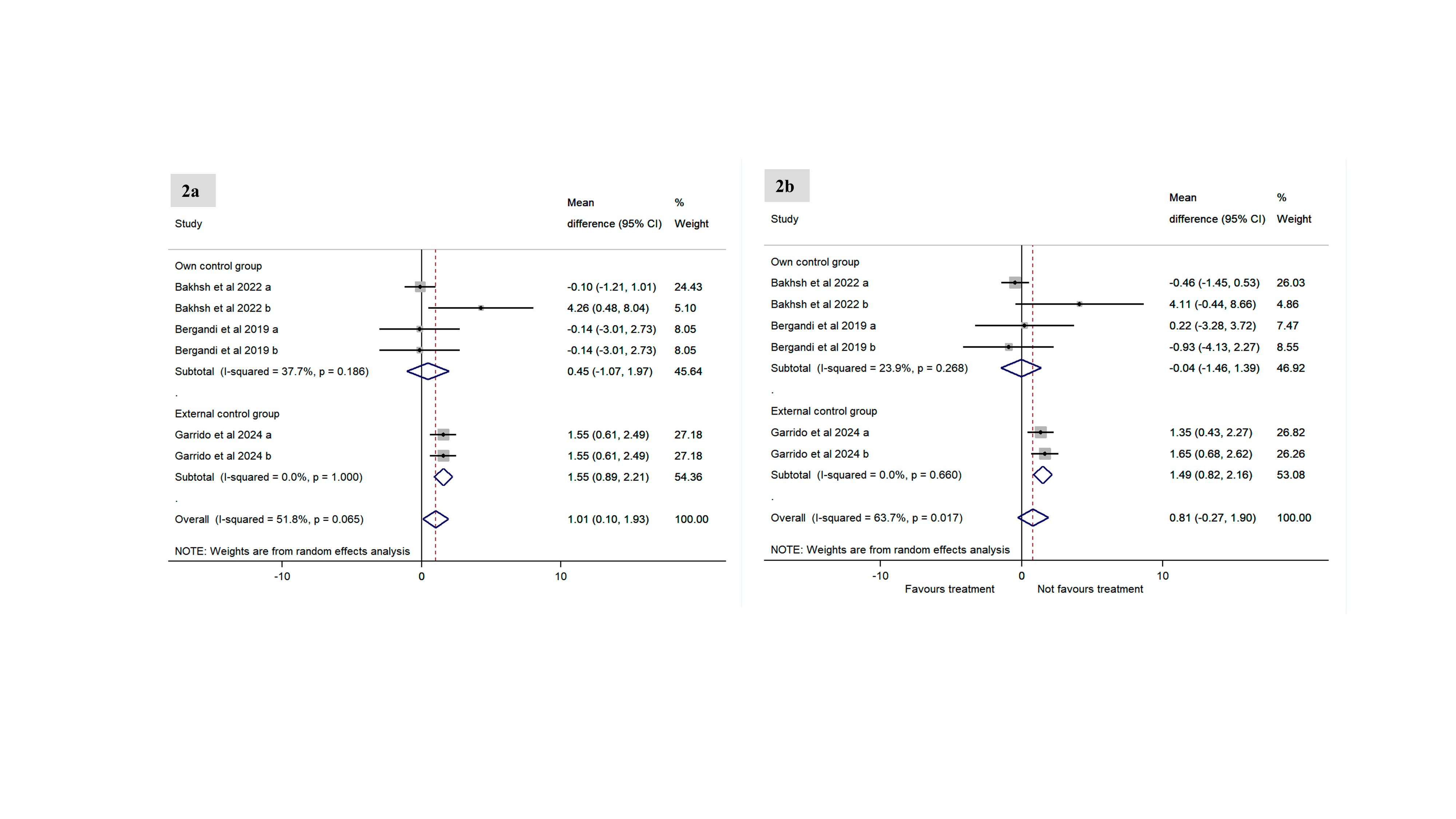

Supplement: Supplementary file 6 — (PNG 660 KB) [file 784_2026_6857_Fig6_ESM.png]

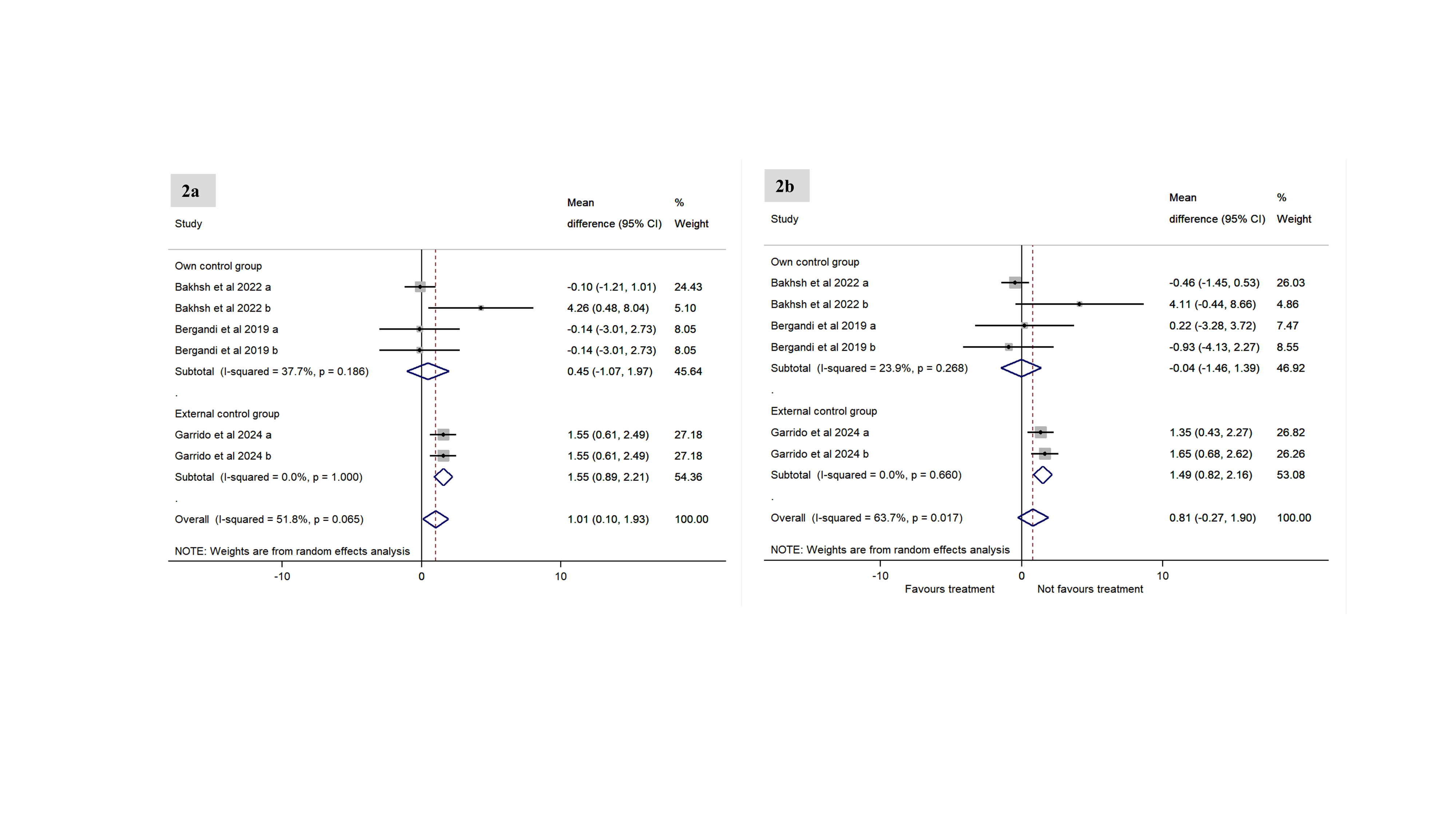

Supplement: Supplementary file 7 — High Resolution Image (TIF 11.8 MB)Supplementary Fig. 2 Subgroup analysis of the effect of endodontic treatment on serum IL-6 levels. 2a. Baseline with external control group. 2b. Post-endodontic treatment with external control group [file 784_2026_6857_MOESM5_ESM.tif]

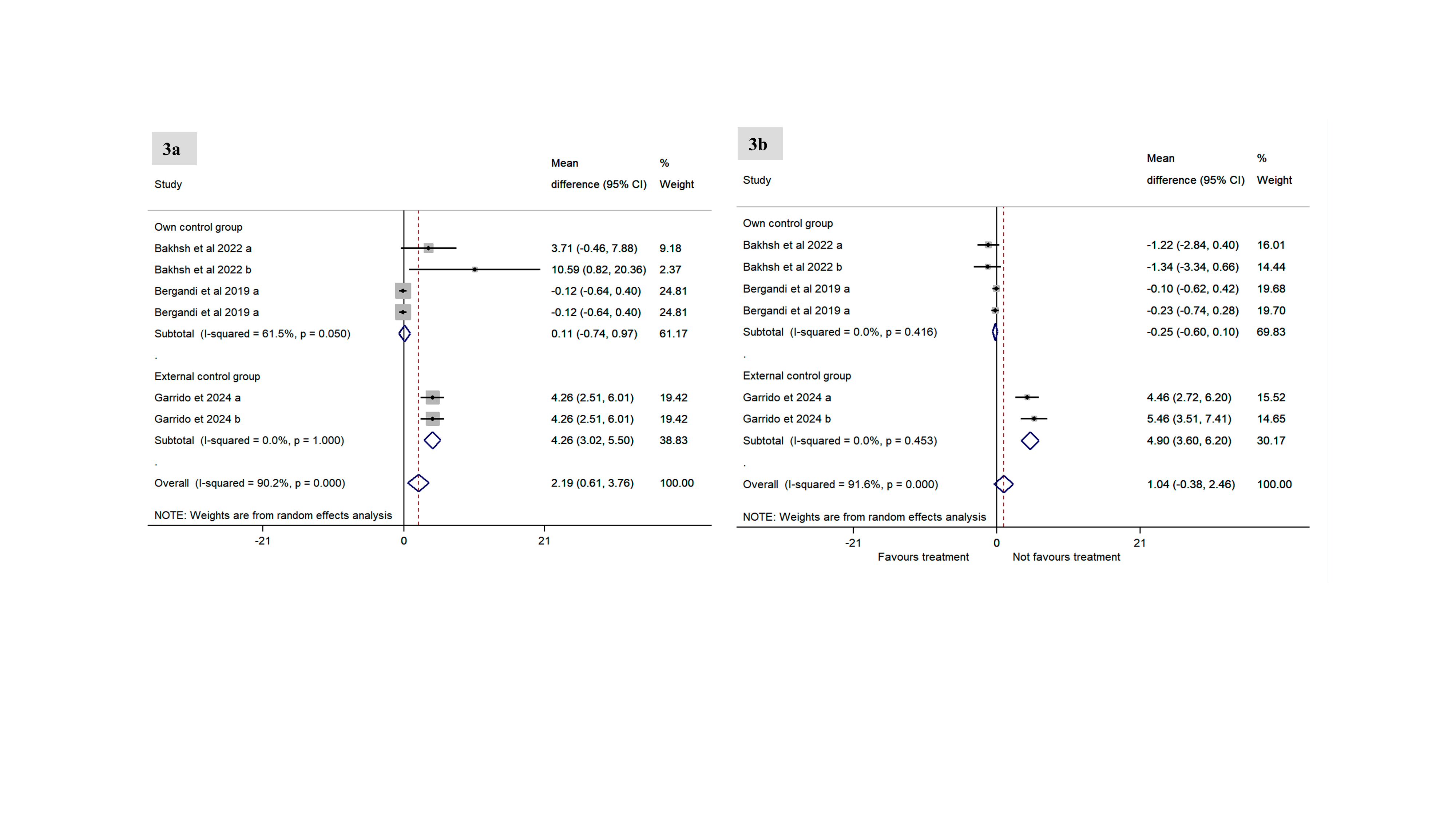

Supplement: Supplementary file 8 — (PNG 664 KB) [file 784_2026_6857_Fig7_ESM.png]

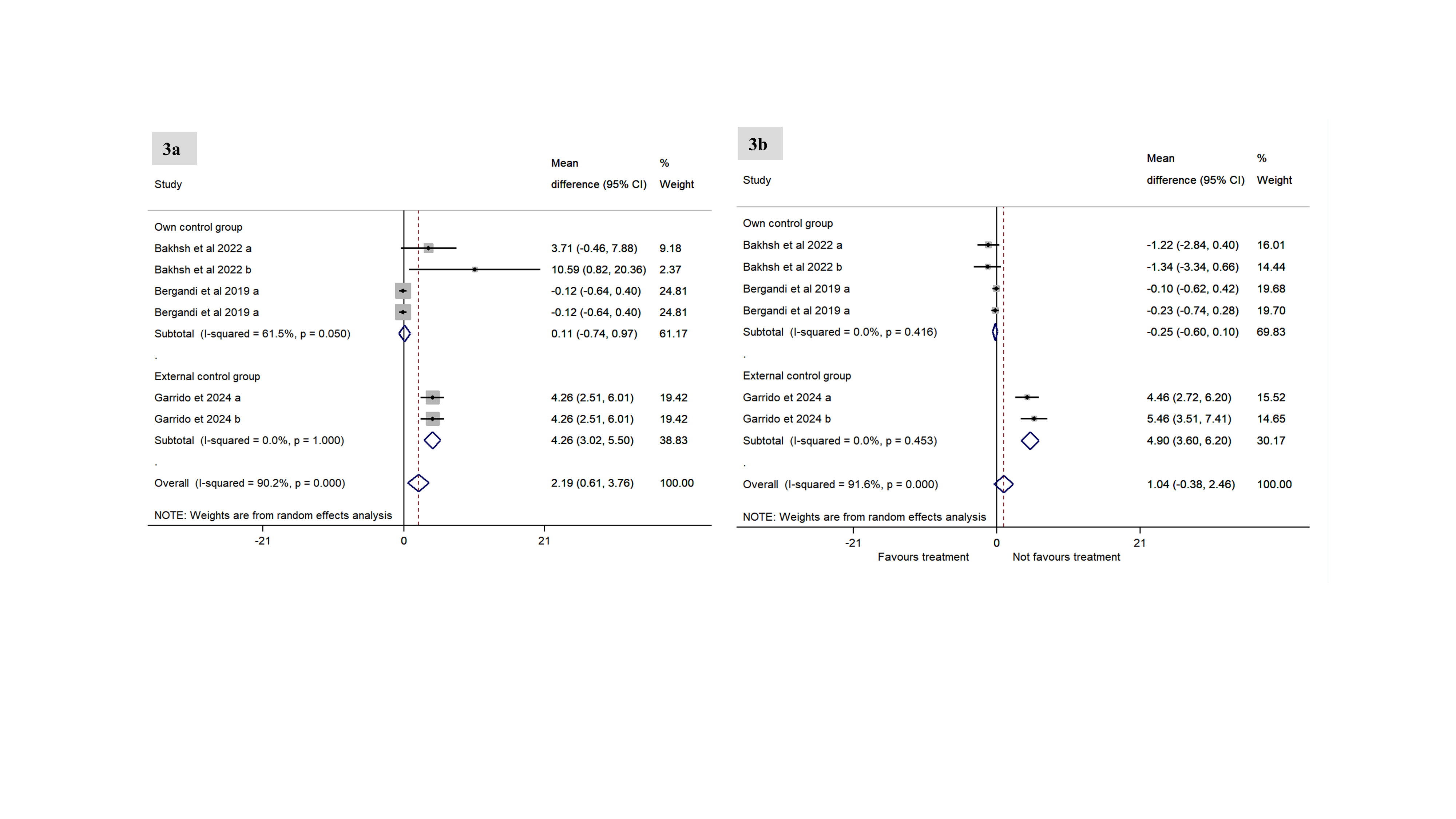

Supplement: Supplementary file 9 — High Resolution Image (TIF 11.8 MB)Supplementary Fig. 3 Subgroup analysis of the effect of endodontic treatment on serum TNF-α levels. 3a. Baseline with external control group. 3b. Post-endodontic treatment with external control group [file 784_2026_6857_MOESM6_ESM.tif]
